# The American Year-Book of Medicine and Surgery

**Published:** 1900-05

**Authors:** 


THE AMERICAN YEAR-BOOK OF MEDICINE AND SURGERY. Being a Yearly Digest of Scientific Progress and Authoritative Opinion in all Branches of Medicine and Surgery, drawn from Journals, Monographs and Text-Books of the Leading American and Foreign Authors and Investigators. Under the general editorial charge of Geo. M. Gould, M.D. Published by W. B. Saunders, Philadelphia. Price, 2 vols., Medicine and Surgery, Cloth, $3 net.

The work before us is the volume on Medicine. This arrangement is a good one, for it was rather confusing under the old system of one volume to have subjects both medical and surgical mixed together. Each division of medicine is presided over by a contributor who is eminently fitted to collaborate and add data on this especial portion of the subject. For instance, we find Dr. Louis Duhring of Philadelphia in charge of the chapter on Dermatology; Dr. Louis Starr has charge of Pediatrics, and so on throughout the domain of medicine and surgery. These men have collaborated only the important advances and given to the busy practitioner the very kernel of all that has been written during the past year in medicine and surgery. This volume is well edited and contains much valuable information. The arrangement is good, print clear, and the binding in keeping with all that comes from the publishing house of W. B. Saunders. R.

---

THE INTERNATIONAL TEXT-BOOK OF SURGERY. By American and British Authors. Edited by J. Collins Warren, M.D., LL.D., Professor of Surgery in Harvard Medical School; Surgeon to the Massachusetts General Hospital; and A. Pearce Gould, M.S., F.R.C.S., Surgeon to Middlesex Hospital, &c. &c. Vol I. General and Special Surgery. Philadelphia: W. B. Saunders, 925 Walnut St. 1900. Price, Cloth, $5.00; Sheep or Half Morocco, $6.00 net.

The ever widening field of Surgery necessitates frequent recasting of older works and the appearance of new ones. The work in point is a highly successful effort to cover the whole subject in a practical manner. The contributors comprise well-known general and special surgeons in America and England and include such names as Golding-Bird, Watson-Cheyne, Fowler, McBurney, M. H. Richardson, McLane Tiffany. Volume I. is devoted to General and Special Surgery. The book is lavishly illustrated. It will no doubt achieve immediate and merited recognition as one of the principal additions to surgical literature of the decade.

---

CHRISTIAN SCIENCE; AN EXPOSITION. A plea for the children and other Helpless Sick. By W. A. Purrington. E. B. Treat & Co., New York.

This volume, with its pathetic and convincing frontispiece, shows with legal succinctness and judicial candor the absurdity, stupidity and danger of this cult of quackery. The book will serve a good purpose in warning against this pseudo-science and increasing prejudice against it. It should be widely read.

---

A CYCLOPEDIA OF PRACTICAL MEDICINE AND SURGERY. A concise Reference Book, alphabetically arranged, of Medicine, Surgery, Obstetrics, Materia Medica, Therapeutics and various Specialties, with particular reference to Diagnosis and Treatment. Compiled under the Editorial Supervision of George M. Gould, M.D., and Walker L. Pyle, M.D., Philadelphia, Pa. P. Blakiston's Sons & Co. 1900.

The great value of this splendid work is that it takes the place in the library of one who has not much to spend on books of many volumes. It is thus put within the reach of many who would otherwise be deprived of much special information. The whole range of medicine is fully covered. It is a mine of information and should be owned by every one who wants a reliable book of reference.

